# Evaluating the response effort and data quality of established political solidarity measures: a pre-registered experimental test in an online survey of the German adult resident population in 2021

**DOI:** 10.1007/s11135-022-01594-4

**Published:** 2023-01-23

**Authors:** Achim Goerres, Jan Karem Höhne

**Affiliations:** Department of Political Science, University of Duisburg-Essen, Duisburg, Germany

**Keywords:** Data quality, Online survey, Political solidarities, Rating scale design, Response behaviour

## Abstract

This experimental study aims to check and improve the quality of 16 established survey measures of political solidarities and related concepts, such as redistribution and social trust. Political solidarities are defined as one’s willingness to share the costs that result from public redistribution that favours people other than oneself and thus constitute a subset of welfare state attitudes. The pre-registered study plan included suggestions for the development of improved rating scales, which we defined as five-point, end verbalized rating scales without non-substantive answer options. The overall results from an experimental online survey in Germany indicate differences in response effort in terms of response times but almost no differences in data quality in terms of criterion validity. Thus, the 16 survey measures show solid instrument validity as well as minor improvements in respondents’ response times. Indeed, the measures are (at least) in the online survey world of Germany of high-quality and warrant inclusion in future surveys with small efficiency gains still attainable.

## Introduction

Improving the measurement of political solidarities, defined as one’s willingness to share the costs that result from public redistribution that favours people other than oneself, is an important endeavour in political science and adjacent research fields. Political solidarities are a multi-dimensional concept whose measurement touches upon the literature on welfare state attitudes and deservingness, forming a part of a broader measure of social cohesion: One’s willingness can be influenced by perceptions of the target group of redistribution (e.g. children, immigrants, or the unemployed), perceptions of the system of redistribution (e.g. local, regional, national, or supranational), and perceptions of the political system’s effectiveness (e.g. political trust) (Goerres 2021).

It is of particular importance to measure political solidarities in a methodologically sound way. First, there is a large and growing literature on welfare state attitudes in political science, sociology and economics, some studies of which may use survey measures of questionable quality (see, for example, Lundmark et al. 2016). In the three decades since 1991, the mention of welfare state attitudes as a main topic of a research article has more than tripled in social science abstracts^1^ (1991–2000: 86, 2001–2010: 161, 2011–2020: 321). Second, some survey measures of political solidarities, such as whether the state should redistribute from the rich to the poor, have become standard questions (or items) in general social surveys with somewhat unclear benefits regarding their validity.^2^ Third, many advanced industrial democracies have to deal with various large-scale changes, some of them exogenous shocks, to their welfare state systems, such as mass immigration, the rise of the populist right, growing inequality, economic crises, or the Covid-19 pandemic. These changes have the potential to undermine the underlying social contract between the citizens and the state that allows extensive redistribution within the welfare state. Governments are becoming aware of these changes and are reacting to them by turning to academia for answers. For example, the German Parliament (Deutscher Bundestag) decided to install a federal institute for social cohesion whose objective is to advise the government on how to deal with the changed situation (original decision on 10th November 2016; funding started in 2018). Finally, and probably most importantly, changes seem to be taking place in the latent cognitive maps of citizens in Europe in their relationship to the state with increasing emphasis on how much is redistributed and how and to whom that redistribution should take place (Cavaillé and Trump 2015).

The quantitative measurement of political solidarities has often been approached under the heading of measuring welfare state attitudes (Goerres and Prinzen 2012); i.e. individual assessments of welfare state institutions, policies and spending therein. Political solidarities are a subset of welfare state attitudes, focusing on the willingness to finance welfare state activities favouring others (Goerres and Tepe 2010). In that literature, certain scales of welfare state attitudes from large international comparative surveys (see, for example, European Social Survey [ESS] and International Social Survey Programme [ISSP]) are commonly used. In our study, we draw upon those scales. The ESS development team, in particular, broadened the measurement of welfare state attitudes immensely in its survey rounds 4 (2008) and 8 (2016). Recently, efforts have been made to expand the types of welfare state services towards which respondents’ attitudes are measured. This is done, for example, by including survey questions on Early Childhood Education policy areas (Neimanns and Busemeyer 2021), confronting respondents with trade-offs between policy areas (Busemeyer and Garritzmann 2017), presenting respondents complex, multidimensional vignettes (Gallego and Marx 2017), or having respondents reveal their concrete willingness to pay for certain policy reforms (Boeri et al. 2001). There is thus an uncoordinated effort and abundance of related attempts to measure political solidarities. What is missing, however, is a concrete quality assessment of established measures in a rigorous measurement set-up focussing on the quality of these scales.

Considering the increasing importance of political solidarity measures for political science and adjacent research fields, in this paper, we experimentally investigate the response effort (response times) and data quality (criterion validity) of existing and newly designed rating scales of survey measures on political solidarities and related concepts. Survey measures of high data quality are a pre-requisite for drawing correct and robust conclusions. We pre-registered our study, including the research questions and analysis plan, at the Open Science Framework.

In what follows, we outline the methodological background on rating scale design and present our research questions. We then describe the experimental design, the survey questions we use, the data collection and study procedure, and the sample characteristics. After that, we present the results of our study and, finally, provide a discussion and conclusion, including perspectives for future research.

## Methodological background and research questions

Numerous national and international social surveys, such as the *CRO*ss *N*ational *O*nline *S*urvey (CRONOS), which is part of the ESS, regularly measure respondents’ attitudes towards and opinions on political solidarities and related concepts, such as redistribution and social trust. In order to measure these constructs, researchers commonly make use of rating scales (i.e., closed answer formats with an ordered list of options). When it comes to rating scales, certain design aspects must be taken into consideration by researchers because these aspects can have a profound impact on respondents’ answer behaviour and thus on response effort and data quality (DeCastellarnau 2018; Krosnick and Presser 2010; Menold and Bogner 2014; Schaeffer and Dykema 2020; Schaeffer and Presser 2003).

For example, decisions must be taken with respect to

1. the scale length (i.e. number of scale points),
2. the scale verbalization (i.e. completely or end verbalized),
3. the inclusion of non-substantive answer options (e.g. “don’t know” or “no opinion”),
4. the scale polarity (i.e. unipolar or bipolar),
5. the inclusion of numeric values (i.e. whether the scale points are provided with or without numbers),
6. the scale direction (i.e. decremental or incremental),
7. and the scale alignment (i.e. horizontal or vertical).

In this study, the first three design aspects—scale length, scale verbalization, and non-substantive answer options—are of primary interest, because research indicates that they have the potential to affect the answer behaviour of respondents. Thus, in this section, we outline the current state of research on these three design aspects.

Based on the range-frequency model by Parducci (1983), scale length is a key aspect of rating scales, because it influences respondents’ understanding of the underlying rating dimension and determines the degree of differentiation (see Menold and Bogner 2014). Literature reviews by Krosnick and Fabrigar (1997) as well as Krosnick and Presser (2010) indicate that five- and seven-point scales work best in terms of reliability and validity (see also DeCastellarnau 2018 for a comprehensive overview). In addition, some evidence suggests that respondents prefer five- and seven-point rating scales over other scale lengths (Krosnick and Fabrigar 1997). One reason for this finding might be that shorter rating scales (less than five points) do not allow sufficient differentiation between answer options, whereas longer rating scales (more than seven points) impede proper differentiation between answer options. However, studies by Tourangeau et al. (2017) as well as Höhne, Krebs, and Kühnel (Under Review) reveal that seven-point rating scales, compared to five-point rating scales, are more prone to primacy effects. Specifically, with seven-point rating scales, respondents’ answers shifted towards the beginning of the rating scale, producing systematic measurement error. Thus, it seems wise to give preference to rating scales with five points rather than with seven points.

Like scale length, scale verbalization is a key aspect to consider when designing rating scales (see DeCastellarnau 2018; Krosnick and Presser 2010; Menold and Bogner 2014; Schaeffer and Dykema 2020; Schaeffer and Presser 2003). The main reason is that verbal labels for all options (i.e., completely verbalized) or only for the end options (i.e., end verbalized) convey crucial information that respondents, being “cooperative communicators” (Schwarz 1996), use in order to understand and answer survey questions meaningfully (Höhne et al. 2021b; Höhne and Yan 2020; Parducci 1983; Sudman et al. 1996; Toepoel and Dillman 2011; Tourangeau 2004; Tourangeau et al. 2007). For example, Höhne et al. (2020, 2021a) compared completely and end-verbalized unipolar and bipolar rating scales with five points. The authors found that end verbalized rating scales perform best in terms of measurement properties, irrespective of scale polarity. Specifically, end verbalized unipolar and bipolar scales result in similar answer distributions, are invariant, and have equidistantly distributed scale points. The authors see the unlabelled centre of the rating scales as responsible for the effect, as they give the impression of equally distanced intervals. Since equidistance is a pre-requisite for the use of rating scales (see Mohler et al. 1998; Rohrmann 1978; Stevens 1946), the use of end verbalized rating scales appears preferable.

Finally, in line with satisficing theory, employing non-substantive answer options may be problematic, because it fosters (strong) satisficing answer behaviour (Krosnick 1991, pp. 219–220). To put it differently, offering non-substantive answer options represents an easy way for respondents to avoid answering survey questions meaningfully. For this reason, some authors recommend not including non-substantive answer options in rating scales (see, for instance, Gilljam and Granberg 1993; Krosnick 1991; Krosnick and Presser 2010; Krosnick et al. 2001; Saris and Gallhofer 2014). Krosnick et al. (2001), for example, analysed data from nine survey experiments investigating the impact of non-substantive answer options on respondents’ answer behaviour. Interestingly, the authors show that the selection of non-substantive answer options was highest among low educated respondents and appears in questions placed towards the end of the survey. They conclude that non-substantive answer options do not improve data quality, but rather preclude the collection of meaningful answers from respondents.

Considering our previously inferred design recommendations with respect to scale length, scale verbalization, and non-substantive answer options it seems best to employ five-point, end verbalized rating scales without non-substantive answer options. First, this scale length produces good data quality and appears to be preferred by respondents. Second, this type of scale verbalization shows good measurement properties in terms of equidistance. Finally, excluding non-substantive answer options may prevent the occurrence of (strong) satisficing answer behaviour.

In this study, we comprehensively searched numerous scientific articles and established social surveys, such as the ESS, for questions addressing political solidarities and related concepts. Based on our search, we compiled a total of 16 survey questions on redistribution, governmental scope, social trust, and welfare chauvinism. The rating scales of these questions varied significantly and, from a methodological perspective, their design might be open to improvement following the previously outlined recommendations. For example, some questions were accompanied by four-point, completely verbalized rating scales with a non-substantive answer option, while some others were accompanied by eleven-point, end verbalized rating scales. In line with the previously outlined design recommendations, we developed five-point, end verbalized rating scales for all survey questions under investigation while maintaining the original question stems and statement formulations. We then conducted a survey experiment in an online access panel in Germany (N = 1513) to systematically test the original and improved rating scales in terms of response effort and data quality. We address the following two research questions:

1. Do the methodologically improved survey questions, compared to the original ones, decrease response effort in terms of response times?
2. Do the methodologically improved survey questions, compared to the original ones, increase data quality in terms of criterion validity?

By addressing these two research questions our study stands out from previous studies for several reasons: (1) much of the existing research was conducted before the emergence of contemporary online surveys (see, for example, DeCastellarnau 2018; Krosnick and Presser 2010), (2) research in this area emphasizes the lack of studies (experimentally) investigating questions on political solidarities and related concepts (Lundmark et al. 2016), (3) the existing research frequently only considers single design aspects, such as polarity (see, for example, Höhne et al. 2020), but does not test multiple design aspects simultaneously, and (4) most studies do not investigate response effort.

## Method

### Experimental design

We used a between-subject design. Respondents were randomly assigned to one out of two experimental groups. The first group (n = 726) received survey questions with rating scales that were taken from established social surveys (original condition). The second group (n = 787) received the same survey questions but with the methodologically improved rating scales (improved condition).

### Questions

*Target questions* We employed 16 target questions that we adopted from scientific articles and established social surveys, such as the ESS. The 16 questions are thematically grouped: redistribution (3 questions), governmental scope (5 questions), social trust (3 questions), and welfare chauvinism (5 questions). For each target question, we developed methodologically improved rating scales (improved condition), while maintaining the phrasing of the original question stems and statement formulations. The 16 target questions were presented at the beginning of the online survey in order to prevent carry-over effects from previous questions. We presented one target question per online survey page (single question presentation). The original German question wordings can be found in the pre-registration on Open Science Framework (see https://osf.io/vzwr3?view_only=fb32a31bf37549daa1192d4501441d12). Appendix 1 shows the English translations of the target questions, including the rating scales, and Appendix 2 displays screenshots of the survey questions.

*Criterion questions:* We used 5 survey questions on political attitudes as criterion measures in order to evaluate criterion validity. For redistribution, governmental scope, and welfare chauvinism, we used one question on the willingness to expend taxpayer money on social benefits and one question on the willingness to facilitate immigration of foreigners. For social trust, we used three questions on political trust (trust in parliament, trust in politicians, and trust in parties). These questions were presented in the third quarter of the survey.

Determining criterion validity is an established method that has been used in previous research (see, for instance, Höhne and Yan 2020; Yeager and Krosnick 2012). The 5 questions were chosen as criterion questions because they are conceptually relevant to the topics of the target questions. In addition, they correlated significantly with all the experimentally manipulated target questions.^3^ In order to determine criterion validity, we investigate which of the two conditions (original or improved) results in higher correlations between the target questions and the criterion questions. Higher (lower) correlations indicate higher (lower) criterion validity. The original German question wordings can be found in the pre-registration on Open Science Framework (see https://osf.io/vzwr3?view_only=fb32a31bf37549daa1192d4501441d12). Appendix 3 shows the English translations of the criterion questions, including rating scales.

### Data collection and study procedures

Data were collected in the Forsa Omninet Panel (omninet.forsa.de) in Germany from 28th July to 16th August 2021. Forsa drew a cross-quota sample from their online panel based on age (young, middle, and old), gender (female and male), and education (low, middle, and high). In addition, they drew quotas based on region (East and West Germany). The quotas were calculated based on the German Microcensus (2019), which served as a population benchmark. The data, including analyses code, are available for replication purposes via the platform Harvard Dataverse (see https://doi.org/10.7910/DVN/XKERRU). This study was pre-registered via the platform Open Science Framework on 27th July 2021.

Forsa invited respondents via email (including two rounds of reminders). The email informed respondents that they would participate in an online survey conducted by the University of Duisburg-Essen (Germany). In addition, it included a link directing respondents to the online survey. On the first page of the online survey, respondents were introduced to the topic (i.e. social and political attitudes) and the procedure of the online survey. Respondents also received a statement of confidentiality assuring them that the study adheres to existing data protection laws and regulations. In addition, the study was approved by the ethics committee of the department of Computer Science and Applied Cognitive Science of the University of Duisburg-Essen (Germany).

We also collected several types of paradata, such as response times, using the open-source tool “Embedded Client Side Paradata (ECSP)” developed by Schlosser and Höhne (2018). Prior informed consent for the collection of paradata was obtained by Forsa as part of the respondents’ registration process. In addition, respondents received modest financial compensation for their participation from Forsa.

Forsa invited a total of 2,848 respondents to participate in the online survey, of which 1115 (39%) did not react to the survey invitation, 168 (6%) were screened out because quotas were already achieved, and 52 (2%) did not finish the online survey. This leaves 1,513 respondents available for statistical analyses (participation rate of about 53% from the panel of volunteers).

### Sample characteristics

Respondents were aged between 18 and 88 years, with a mean age of 52 years (SD = 17 years), and 49% of them were female. In terms of education, 33% completed lower secondary school or less (low education level), 27% intermediate secondary school (medium education level), and 41% college preparatory secondary school or university (high education level).

In order to evaluate the effectiveness of random assignment and the sample composition between the two experimental groups, we conducted several statistical tests. The results revealed no statistically significant differences between the experimental groups with respect to age, gender, and education.

## Results

For comparability, we initially recoded the answer options of the survey questions to a scale running from 0 to 1. This was done for the 16 target questions as well as for the 5 criterion questions used in this study. In all analyses, we use a p-level of 0.05 to determine statistical significance. We used Stata (version 17) for data preparation and analyses.

### Answer distributions

In the first step of our analsis, we investigated the answer distributions of the 16 target questions. Since the rating scales partially differ in length (e.g. eleven points in the original condition and five points in the improved condition), we decided to compare the means of the scales ranging from 0 to 1 instead of proportions. Accordingly, we conducted two-sample Student t-tests.^4^ Table 1 reports the statistical results.

**Table 1:** Mean values of the 16 target questions for each condition (original and improved)

Questions	Original	Improved	Questions	Original	Improved
Red 1	**0.69**	**0.65**	Soc 1	0.43	0.43
Red 2	0.64	0.65	Soc 2	0.55	0.55
Red 3	0.59	0.59	Soc 3	0.49	0.49
Gov 1	0.64	0.63	Wel 1	0.40	0.42
Gov 2	0.47	0.50	Wel 2	0.45	0.47
Gov 3	0.78	0.79	Wel 3	0.44	0.44
Gov 4	0.52	0.51	Wel 4	0.49	0.48
Gov 5	0.63	0.62	Wel 5	0.45	0.44

Bold indicates significant mean differences (*p* < 0.05). *red* Redistribution, *gov* Governmental scope, *soc* Social trust, *wel* Welfare chauvinism

The results in Table 1 show that the mean differences between the original and improved conditions are negligibly small (differences < 0.05). This applies to all target questions, irrespective of the concepts (i.e. redistribution, governmental scope, social trust, and welfare chauvinism). The only exception is the first question on redistribution (red 1), which has a significantly higher mean value in the original condition. Nonetheless, these results provide strong empirical evidence that respondents’ answer behaviour is not affected by the rating scale design when respondents are asked survey questions on political solidarities and related concepts.

### Response times

Response times enjoy a long tradition in social psychology and survey research (Couper and Kreuter 2013; Yan and Tourangeau 2008) and have proven to be useful measures of response effort (Bassili and Scott 1996; Fazio 1990; Höhne et al. 2017; Lenzner et al. 2010; Yan and Olson 2013). In general, researchers assume that the time taken to process questions corresponds (directly) to the response effort required to answer a survey question. This, in turn, suggests that the longer (shorter) a respondent needs to answer a question, the higher (lower) the response effort.

We investigated the response effort associated with the survey questions between the original and improved conditions. The response times were measured in milliseconds and were defined as the time elapsing between the presentation of the question on the screen and the submission of the online survey page. To compare response times, we computed median values and thus used Mann–Whitney (U) tests. Table 2 reports the statistical results.

**Table 2:** Median response times (ms) of the 16 target questions for each condition (original and improved)

Questions	Original	Improved	Questions	Original	Improved
Red 1	15,242	14,649	Soc 1	**13,770**	**12,515**
Red 2	**16,153**	**14,116**	Soc 2	**12,168**	**11,374**
Red 3	**28,707**	**26,834**	Soc 3	**11,155**	**10,416**
Gov 1	**18,417**	**17,411**	Wel 1	**12,404**	**11,084**
Gov 2	**11,390**	**10,362**	Wel 2	**9,319**	**8,454**
Gov 3	**10,005**	**8,988**	Wel 3	7,802	7,370
Gov 4	**10,518**	**9,702**	Wel 4	7,103	6,971
Gov 5	**8,998**	**8,205**	Wel 5	**6,973**	**6,301**

Bold indicates significant median differences (*p* < 0.05). *red* Redistribution, *gov* Governmental scope, *soc* Social trust, *wel* Welfare chauvinism

Comparing median, as displayed in Table 2, one can observe that respondents take a consistently longer time to answer the survey questions with the original rating scales than the survey questions with the methodologically improved rating scales. Specifically, we find significantly longer median response times in the original condition for 13 out of 16 comparisons. The only exceptions are the first question on redistribution (red 1) as well as the third and fourth questions on welfare chauvinism (wel 3 and 4), for which we do not find significant differences. Overall, these findings provide strong empirical evidence that the methodologically improved questions, compared to the original questions, require less response effort in terms of response times.

### Criterion validity

Finally, we investigated data quality in terms of criterion validity between the original and improved conditions. Specifically, we examined the strength of the correlations between the 16 experimentally manipulated target questions and the five criterion questions on social benefits, immigration, and political trust (i.e. trust in parliament, trust in politicians, and trust in parties). Remember that higher (lower) correlations indicate higher (lower) criterion validity. The criterion validity analyses were conducted by estimating unstandardized OLS regression coefficients with the target questions as independent variables and the criterion questions as dependent variables. Table 3 reports the statistical results.

**Table 3:** OLS regressions to determine criterion validity (unstandardized coefficients)

Social benefits	Original	Improved	Immigration	Original	Improved
Red 1	**0.45**	**0.31**	Red 1	–0.24	–0.20
Red 2	0.38	0.30	Red 2	–0.19	–0.15
Red 3	0.36	0.30	Red 3	–0.20	–0.14

Social benefits			Immigration		
Gov 1	0.13	0.16	Gov 1	-	-
Gov 2	0.32	0.31	Gov 2	–0.23	–0.31
Gov 3	0.16	0.11	Gov 3	-	-
Gov 4	0.48	0.47	Gov 4	–0.35	–0.35
Gov 5	0.41	0.38	Gov 5	–0.24	–0.24

Trust in parliament			Trust in politicians		
Soc 1	**0.34**	**0.23**	Soc 1	0.26	0.24
Soc 2	0.34	0.27	Soc 2	0.29	0.26
Soc 3	0.23	0.20	Soc 3	0.22	0.20

Trust in parties					
Soc 1	0.24	0.21			
Soc 2	0.24	0.22			
Soc 3	0.17	0.17			

Social benefits			Immigration		
Wel 1	0.37	0.35	Wel 1	–0.60	–0.56
Wel 2	0.37	0.36	Wel 2	–0.59	–0.54
Wel 3	0.49	0.43	Wel 3	–0.46	–0.48
Wel 4	0.47	0.43	Wel 4	–0.42	–0.45
Wel 5	0.53	0.52	Wel 5	–0.39	–0.35

Bold indicates significant differences (*p* < 0.05). *red* Redistribution, *gov* Governmental scope, *soc* Social trust, *wel* Welfare chauvinism. We tested 16 target questions: red 1–3, gov 1–5, soc 1–3, and wel 1–5. The experimentally manipulated target questions on governmental scope (gov 1 and 3) did not correlate significantly with the criterion question on immigrants. Therefore, we do not report their regression coefficients

As shown in Table 3, the original questions on redistribution, governmental scope, social trust, and welfare chauvinism show slightly higher correlations with the criterion questions than their improved counterparts. There are only a few exceptions, such as the second governmental scope (gov 2) question on immigration. Even though the majority of the original questions show higher correlations, only two comparisons show significant differences: (1) the first redistribution question (red 1) on social benefits and (2) the first social trust question (soc 1) on trust in parliament. For the remaining comparisons, no significant differences exist. Altogether, these findings indicate that the original and methodologically improved questions have similar levels of data quality in terms of criterion validity.

## Discussion and conclusion

The aim of this experimental study was to evaluate the response effort and data quality of established political solidarity measures, a sub set of welfare state attitudes. Response effort was measured using response times (Bassili and Scott 1996; Fazio 1990; Höhne et al. 2017; Lenzner et al. 2010; Yan and Olson 2013), whereas data quality was determined by estimating criterion validity (see, for instance, Höhne and Yan 2020; Yeager and Krosnick 2012). The results indicate differences in response time, but almost no differences in criterion validity. In the following, we discuss the empirical findings in detail.

In the first step of our analysis, we investigated the answer distributions of the questions with the original and improved rating scales. The mean comparisons revealed almost no differences between the two conditions, even though the design of the rating scales differed substantially in some cases (e.g. four-point, completely labelled rating scales with a non-substantive answer option vs. five-point, end verbalized rating scales without non-substantive answer option). We see these findings as good news, because they show that established measures of political solidarities and related concepts are robust against rating scale effects. To put it differently, respondents’ answer behaviour is not affected by the rating scales we examined.

In order to evaluate the response effort of the questions with the original and improved rating scales, we collected response times in milliseconds (i.e., the time elapsing between the presentation of the question on the screen and the submission of the online survey page). In doing so, we followed a long line of research in social psychology and survey research (Couper and Kreuter 2013; Yan and Tourangeau 2008). Our findings indicated substantial differences between the two rating scale conditions. Response times were consistently higher in the original condition than they were in the improved condition. This finding points to the fact that the questions with the improved rating scales, compared to the questions with the original rating scales, reduce response effort. Following Bradburn (1978), we argue that it is the responsibility of researchers not to gratuitously increase response effort for respondents; i.e., if this increase is not expected to improve data quality. We thus recommend the use of the improved rating scale design in place of the original rating scale designs.

To evaluate data quality, we examined the criterion validity of the questions with the original rating scales and their improved counterparts. Specifically, we determined the strength of the associations between the experimentally manipulated target questions and the criterion questions that all respondents were asked. The results demonstrated almost no criterion validity differences between the two rating scale conditions, which indicates that the original and improved rating scales do not differ in data quality. Even though the original rating scales do not follow contemporary best practices, they can be considered equal in data quality to the improved rating scales. In our opinion, this is also good news, as it suggests that existing measures of political solidarities and related concepts are of good data quality in terms of criterion validity.

This study has some limitations that provide avenues for future research. First and foremost, we conducted our study in one country (Germany). However, some of the questions under investigation in this study were selected from cross-cultural and cross-national surveys, such as the ESS. We therefore cannot draw any conclusions beyond Germany and thus we call for further cross-cultural and cross-national research. Second, and relatedly, we used a quota sample from a non-probability access panel. This does not decrease the internal validity of our experimental study, but it might limit the generalizability of our empirical findings. Hence, it would be worthwhile to investigate rating scale design of questions on political solidarities and related concepts using a probability-based sample to increase generalizability. Third, since respondents of this study were members of an access panel who participate in web surveys on a regular basis, they may have a high level of survey experience compared to the general population. Some research indicates that respondents with high survey experience differ from respondents with low survey experience in terms of response behaviour (Toepoel et al. 2008). For this reason, we recommend that future studies take respondents’ level of survey experience into account.

Despite its limitations, this study provides important insights on the impact of rating scale design on answer behaviour. Keeping in mind both our findings and the contemporary best practices on rating scale design we believe that a methodologically sound rating scale has the following characteristics: five-point, end verbalized, and without non-substantive answer options. This applies, at least, to measuring political solidarities and related concepts. The improved rating scale design results in a level of data quality that is comparable to the original rating scale designs, but requires less response effort.

## Figures and Tables

**Fig. 1:**
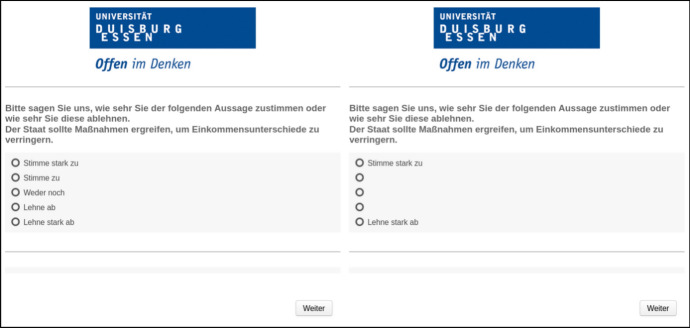
Example screenshots of the first survey question on redistribution. *Note* Original condition on the left and improved condition on the right

## Data Availability

For replication purposes, data is available to the scientific community via Harvard Dataverse (see https://dataverse.harvard.edu/dataset.xhtml?persistentId=doi:10.7910/DVN/XKERRU).

## Appendix 1

English translations of the 16 target questions used in this study (original condition only).

1. To what extent do you agree or disagree with the following statement? The state should take measures to reduce income inequality. (redistribution 1)
  Rating scale: 1 “Agree strongly”, 2 “Agree”, 3 “Neither/nor”, 4 “Disagree”, and 5 “Disagree strongly”
2. Now please indicate to what extent the following things should be the responsibility of the state. The state should reduce the income gap between rich and poor. (redistribution 2)
  Rating scale: 1 “Responsible in any case”, 2 “Responsible”, 3 “Not responsible”, 4 “Definitely not responsible”, and 5 “Can’t say”
3. Here are two statements about a controversial issue and a scale that you can use to grade your own opinion about it. If you completely agree with the statement above the scale, select the answer box at the top. If you completely agree with the statement below the scale, select the answer box at the bottom. If your opinion is somewhere in between, you can express this with one of the answer boxes in between. (redistribution 3)
  Rating scale: 1 “The state should take more responsibility for ensuring that every citizen is covered” to 11 “Individual citizens should take more responsibility for themselves”
4. People have different ideas about what the state should and should not be responsible for. For each of the following tasks, please tell us how much the state should be responsible for. Should the state be responsible for ensuring a decent standard of living in old age? (governmental scope 1)
  Rating scale: 1 “The state should not be responsible for this at all” to 11 “The state should be fully responsible for this”
5. People have different ideas about what the state should and should not be responsible for. For each of the following tasks, please tell us how much the state should be responsible for. Should the state be responsible for ensuring a decent standard of living in young age? (governmental scope 2)
  Rating scale: 1 “The state should not be responsible for this at all” to 11 “The state should be fully responsible for this”
6. People have different ideas about what the state should and should not be responsible for. For each of the following tasks, please tell us how much the state should be responsible for. Should the state be responsible for ensuring childcare options for working parents? (governmental scope 3)
  Rating scale: 1 “The state should not be responsible for this at all” to 11 “The state should be fully responsible for this”
7. People have different ideas about what the state should and should not be responsible for. For each of the following tasks, please tell us how much the state should be responsible for. Should the state be responsible for ensuring a decent standard of living for the unemployed? (governmental scope 4)
  Rating scale: 1 “The state should not be responsible for this at all” to 11 “The state should be fully responsible for this”
8. People have different ideas about what the state should and should not be responsible for. For each of the following tasks, please tell us how much the state should be responsible for. Should the state be responsible for ensuring a decent standard of living for poor people? (governmental scope 5)
  Rating scale: 1 “The state should not be responsible for this at all” to 11 “The state should be fully responsible for this”
9. In general, do you think that most people can be trusted, or that you can't be careful enough when dealing with other people? (social trust 1)
  Rating scale: 1 “You can't be too careful” to 11 “Most people can be trusted”
10. Do you think most people try to take advantage of you when they have the opportunity, or do most people try to be fair? (social trust 2)
  Rating scale: 1 “Most people try to take advantage of me” to 11 “Most people try to behave fair”
11. Do you think that people mostly try to be helpful, or that people mostly look out for their own advantage? (social trust 3)
  Rating scale: 1 “People are mostly looking out for their own advantage” to 11 “People mostly try to be helpful”
12. Immigrants from outside the EU should have less entitlement to social welfare in the future than people born in Germany. (welfare chauvinism 1)
  Rating scale: 1 “Strongly agree” to 5 “Strongly disagree”, and “Don’t know”
13. Immigrants from the EU should have less entitlement to social welfare in the future than people born in Germany. (welfare chauvinism 2)
  Rating scale: 1 “Strongly agree” to 5 “Strongly disagree”, and “Don’t know”
14. The welfare state makes people lazy. (welfare chauvinism 3)
  Rating scale: 1 “Strongly agree” to 5 “Strongly disagree”, and “Don’t know”
15. Because of the welfare state, people no longer take care of themselves. (welfare chauvinism 4)
  Rating scale: 1 “Strongly agree” to 5 “Strongly disagree”, and “Don’t know”
16. The state should increase social benefits. (welfare chauvinism 5)
  Rating scale: 1 “Strongly agree” to 5 “Strongly disagree”, and “Don’t know”

*Note* In the improved condition, we adopted the original rating scales, but transformed them into five-point, end verbalized rating scales (without non-substantive answer options). The order of the questions in the online survey corresponds to the presentation order in Appendix 1. We presented one question per online survey page (single question presentation). The original German question wordings can be found in the pre-registration on Open Science Framework (see https://osf.io/vzwr3?view_only=fb32a31bf37549daa1192d4501441d12). Red 1 was adopted from the ESS (Round 1), red 2 was adopted from the ISSP (2006), red 3 was adopted from the WVS (Wave 2), gov 1, gov 3, and gov 4 were adopted from the ESS (Round 4), soc 1 to 3 were adopted from the ESS (Round 4), and wel 1 to 5 were adopted from de Koster et al. (2013). Gov 2 and 5 were self-created and inspired by questions from the ESS (Round 4).

## Appendix 2

Example screenshots of the first survey question on redistribution.

See Fig. 1.

## Appendix 3

English translations of the 5 criterion questions used in this study.

1. Now let us deal with at some political issues. Some people want fewer taxes and contributions, even if that means fewer social benefits, while other people want more social benefits, even if that means more taxes and contributions. What is your position on the issue of taxes and social benefits? (social benefits)
  Rating scale: 1 “Fewer taxes and contributions, even if that means fewer social benefits” to 11 “More social benefits, even if that means more taxes and contributions”
2. Now we are talking about immigration opportunities for foreigners. Should immigration opportunities for foreigners be facilitated or restricted? (immigration)
  Rating scale: 1 “Immigration opportunities for foreigners should be facilitated” to 11 “Immigration opportunities for foreigners should be restricted”
3. Please tell us for each public institution or group how much you personally trust each of them. How about …
4. the parliament? (trust in parliament)
5. the politicians? (trust in politicians)
6. the parties? (trust in parties)

Rating scale: 1 “I do not trust at all” to 11 “I trust completely”

*Note* The order of the questions in the online survey corresponds to the presentation order in Appendix 3. The questions on social benefits and immigration were presented individually (single question presentation) and the three questions on political trust (i.e., trust in parliament, trust in politicians, and trust in parties) were presented on one online survey page (multiple question presentation). The original German question wordings can be found in the pre-registration on Open Science Framework (see https://osf.io/vzwr3?view_only=fb32a31bf37549daa1192d4501441d12).

## Footnotes

1 Search results in the Social Sciences Citation Index (SSCI) database on the query (“welfare state” OR “social policy”) & attitud*).

2 The survey question was asked first in 1981 in the section “Role of Government I” of the International Social Survey Programme (ISSP).

3 The only exceptions are questions on governmental scope (gov 1 and 3).

4 We conducted Levene-tests to determine variance differences between the original and improved conditions. We conducted the t-tests in correspondence to the outcome of the Levene-tests.

## Code availability

For replication purposes, code is available to the scientific community via Harvard Dataverse (see https://dataverse.harvard.edu/dataset.xhtml?persistentId=doi:10.7910/DVN/XKERRU).
